# Amoxicillin-Clavulanate-Induced Pityriasis Rosea: A Pharmacovigilance Case Report

**DOI:** 10.7759/cureus.102309

**Published:** 2026-01-26

**Authors:** Cecil Franz, Divya Handa, Girish Joseph, Neena Bhatti, Dinesh K Badyal

**Affiliations:** 1 Pharmacology, Christian Medical College & Hospital, Ludhiana, IND

**Keywords:** adverse cutaneous drug reaction, amoxicillin–clavulanate, causality assessment, drug-eruption, pharmacovigilance program of india, pityriasis rosea

## Abstract

Adverse drug reactions (ADRs) are a significant cause of morbidity and represent an important public health concern. Cutaneous adverse drug reactions (CADRs) are the most frequently encountered form of ADRs, with antibiotics being among the leading causative drug classes. Although amoxicillin-clavulanate is widely regarded as a safe and commonly prescribed antibiotic, rare and atypical cutaneous reactions may occur and pose diagnostic challenges.

We report a rare case of amoxicillin-clavulanate-induced pityriasis rosea in a 34-year-old male who presented with a progressive pruritic and painful flexural rash following antibiotic therapy for an upper respiratory tract infection. The eruption was initially misdiagnosed as varicella and scabies, leading to delayed appropriate management. A dermatological evaluation established the diagnosis of drug-induced pityriasis rosea. Prompt discontinuation of the offending drug and initiation of topical corticosteroids and antihistamines resulted in complete clinical resolution. Causality assessment using the Naranjo Adverse Drug Reaction Probability Scale indicated a probable association, while severity and preventability assessments classified the reaction as moderate and probably preventable, respectively. This case highlights the importance of maintaining a high index of suspicion for drug-induced dermatoses, particularly with commonly prescribed antibiotics, and underscores the value of pharmacovigilance reporting in improving drug safety.

## Introduction

Adverse drug reactions (ADRs) are defined by the World Health Organization as “a response to a medication that is noxious and unintended and occurs at doses normally used in man” [1]. ADRs represent a significant cause of morbidity and healthcare burden, adversely affecting patient safety and quality of life [2]. Among these, cutaneous adverse drug reactions (CADRs) are the most frequently encountered in clinical practice [3].

Antibiotics are among the leading drug classes implicated in CADRs, accounting for nearly 30-50% of reported cases [4]. Penicillins and cephalosporins are the most common offenders, while sulfonamides and fluoroquinolones are more often associated with severe cutaneous reactions. Antibiotic-induced skin manifestations range from mild eruptions such as maculopapular rashes and urticaria to serious, potentially life-threatening conditions, including Stevens-Johnson syndrome (SJS) and toxic epidermal necrolysis (TEN) [4].

Amoxicillin-clavulanate is a widely prescribed antibiotic combination in both primary care and emergency settings. Amoxicillin is a semi-synthetic, acid-stable β-lactam antibiotic, while clavulanic acid acts as a β-lactamase inhibitor, extending antimicrobial coverage to β-lactamase-producing organisms [5-7]. This combination is commonly used to treat respiratory tract infections, urinary tract infections, skin and soft tissue infections, meningitis, and other bacterial infections [6]. Despite their broad use and favorable safety profile, β-lactam antibiotics are known to cause both immediate and delayed hypersensitivity reactions [8]. Amoxicillin-clavulanate is generally well tolerated, with gastrointestinal adverse effects being the most commonly reported [9]. Dermatological adverse effects are relatively uncommon and are typically mild; however, rare severe reactions such as anaphylaxis, SJS, and TEN have been described [10].

Pityriasis rosea is a rare cutaneous adverse reaction associated with amoxicillin-clavulanate. Data from the World Health Organization pharmacovigilance database accessed through VigiAccess report 222,722 ADRs linked to this drug combination, of which 112,654 (approximately 37%) involve skin and subcutaneous tissue disorders. Only seven cases have been reported as pityriasis rosea, underscoring the rarity of this presentation [11]. Given the widespread and often empirical use of amoxicillin-clavulanate, awareness of rare and atypical cutaneous adverse reactions is essential for early recognition and appropriate management. This case report aims to highlight an uncommon presentation of pityriasis rosea induced by amoxicillin-clavulanate and to contribute to existing pharmacovigilance data.

## Case presentation

A 34-year-old male with no known comorbidities, no prior history of drug allergies or ADRs, and not on any long-term medications presented with a skin eruption. He had no previous similar episodes and no relevant medical or family history. He had been prescribed a fixed-dose combination of amoxicillin-clavulanate for an upper respiratory tract infection. There was no prior history of drug allergy, atopy, recent vaccination, or viral illness. Approximately 48 hours after initiating therapy, the patient developed an erythematous rash localized to the axillary region. Over the next two days, the eruption gradually progressed to involve flexural areas, including the cubital fossae and groin. The lesions were associated with intense pruritus. There was no associated fever, mucosal involvement, vesiculation, or systemic symptoms.

The patient initially consulted a physician and was clinically diagnosed with varicella (chickenpox), for which oral antiviral therapy was prescribed. However, the patient did not initiate treatment and sought a second medical opinion. At that consultation, the eruption was diagnosed as scabies, and topical therapy was prescribed. Following topical treatment, the patient noted worsening of the rash, with increased erythema, pruritus, and discomfort.

Subsequently, the patient consulted a dermatologist. Cutaneous examination revealed multiple well-defined, oval erythematous plaques with mild peripheral scaling, involving a flexural area. The lesions appear symmetrical, slightly raised, and non-vesicular, with no evidence of ulceration, crusting, or mucosal involvement. The overall morphology and distribution are suggestive of a pityriasis rosea-like eruption, particularly in a drug-related context. Amoxicillin-clavulanate was identified as the probable offending agent.

**Figure 1:**
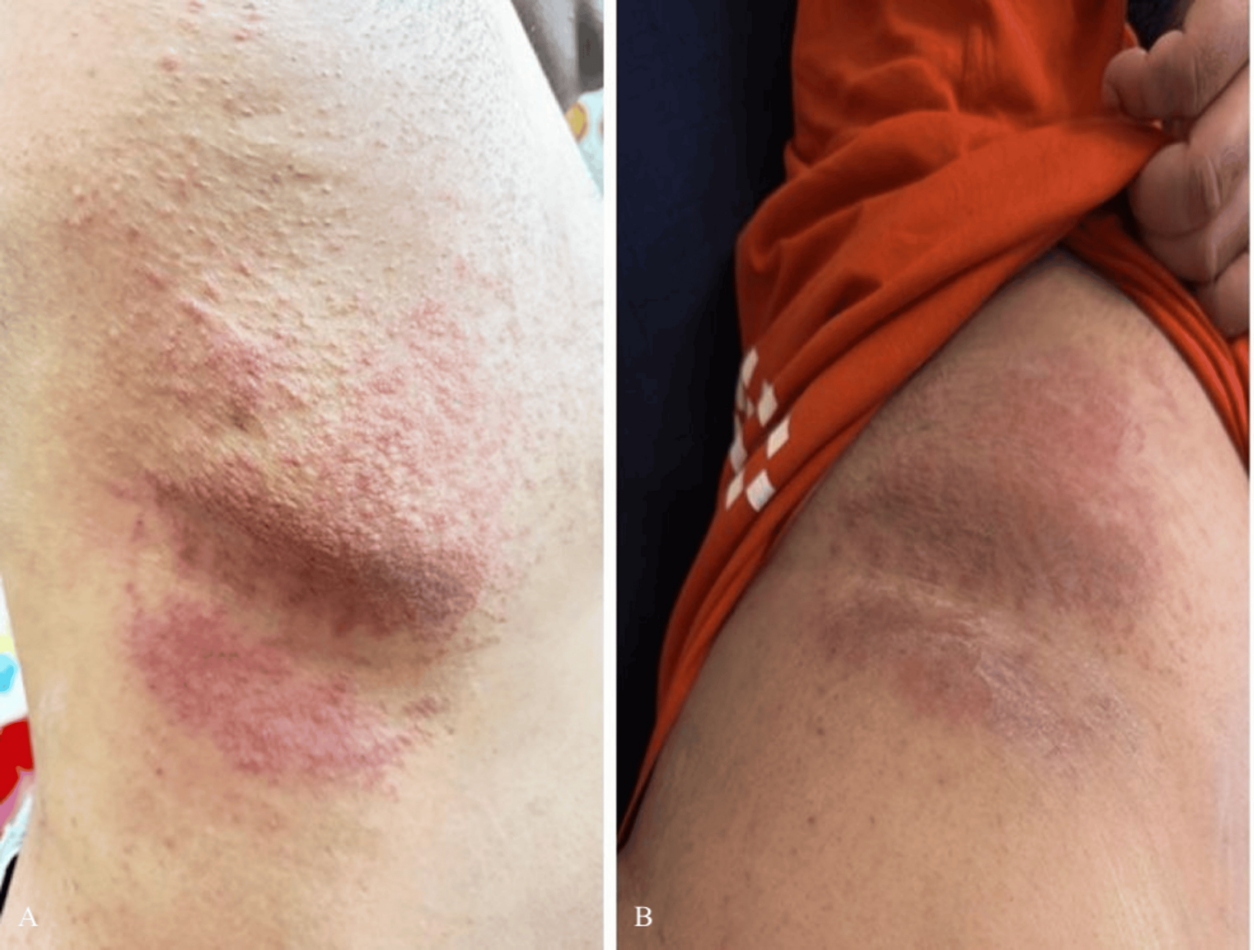
Presentation of pityriasis rosea following the administration of suspect product A: Erythematous oval-to-round plaques with peripheral scaling involving the axillary region and adjacent flexural areas, consistent with pityriasis rosea at presentation following amoxicillin-clavulanate exposure. B: Progression of pityriasis rosea lesions involving flexural regions, showing multiple erythematous plaques with scaling and surrounding inflammation prior to discontinuation of the offending drug.

The lesions with which the patient presented are shown in Figure 1.

**Figure 2:**
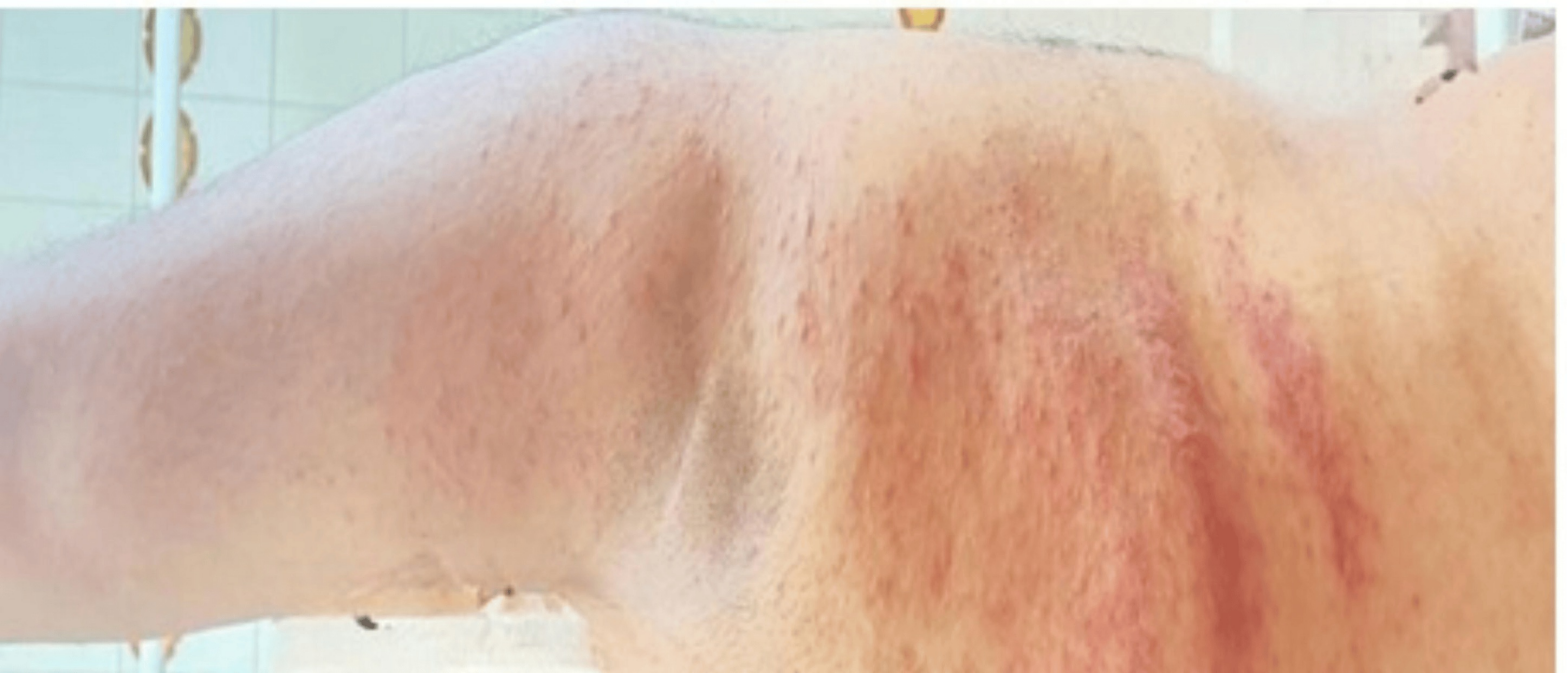
Post-drug discontinuation Complete resolution of cutaneous lesions following withdrawal of amoxicillin-clavulanate and initiation of topical corticosteroids and antihistamines, demonstrating clinical recovery.

The suspected drug was immediately discontinued, and the patient was started on topical corticosteroids and oral antihistamines. Over the next 10-14 days, the patient demonstrated marked clinical improvement, with resolution of pruritus and pain and gradual clearing of lesions. No recurrence or post-inflammatory complications were observed on follow-up. This is shown in Figure 2.

Causality, severity, and preventability assessment

Causality assessment was performed using the Naranjo Adverse Drug Reaction Probability Scale, which yielded a score of 6, indicating a probable ADR. This assessment was based on the temporal relationship between drug exposure and symptom onset, clinical improvement following drug withdrawal, and exclusion of alternative etiologies. The Naranjo probability scale consists of 10 structured questions, each answered as “yes,” “no,” or “don’t know,” with corresponding scores of -1, 0, +1, or +2 assigned based on the response. The cumulative score is then used to determine the likelihood of an ADR: definite (≥9), probable (5-8), possible (1-4), or doubtful (≤0). The total score can range from -4 to +13 [12].

A detailed summary of ADR assessment is provided in Table 1, where causality was assessed using the Naranjo Adverse Drug Reaction Probability Scale, severity was graded according to the Modified Hartwig and Siegel Severity Assessment Scale, and preventability was evaluated using the Schumock and Thornton criteria [13,14]. A summary of ADR assessment using standardized pharmacovigilance tools is shown in Table 1.

**Table 1:** Summary of adverse drug reaction assessment using standardized pharmacovigilance tools

Assessment Tool	Key Criteria Applied	Results	Interpretation
Naranjo Adverse Drug Reaction Probability Scale	Temporal relationship with drug intake, improvement after drug withdrawal, absence of alternative causes	Score = 6	Probable adverse drug reaction
Modified Hartwig and Siegel Severity Assessment Scale	Drug discontinuation required; active medical treatment needed; no hospitalization or permanent harm	Level 3	Moderate severity adverse drug reaction
Schumock and Thornton Preventability Scale	Availability of alternative antibiotics; non-essential use of the implicated drug for mild infection	Yes	Probably preventable adverse drug reaction

The ADR was reported to the institutional Adverse Drug Monitoring Centre under the Pharmacovigilance Programme of India (PvPI).

## Discussion

Pityriasis rosea is an acute, self-limited papulosquamous dermatosis that predominantly affects children and young adults. It classically begins with a “herald patch,” followed by multiple oval erythematous scaly lesions distributed along Langer’s lines of cleavage on the trunk and proximal extremities, producing the characteristic “Christmas tree” pattern. Typical lesions are dull pink to salmon-colored, oval or elliptical macules measuring 0.5-1 cm, with a peripheral collarette of scale [15]. Drug-induced pityriasis rosea-like eruptions differ from classical pityriasis rosea in several respects, including atypical morphology, increased pruritus, absence of a herald patch, and a clear temporal relationship with drug exposure [16]. In the present case, the rapid onset of lesions within 48 hours of initiating amoxicillin-clavulanate, continued progression despite symptomatic treatment, and prompt improvement following drug withdrawal strongly support a drug-induced etiology.

Misdiagnosis of CADRs is common, particularly when clinical presentations resemble infectious dermatoses. In this case, the eruption was initially misdiagnosed as varicella and later as scabies, resulting in delayed appropriate management and worsening symptoms. Similar diagnostic challenges have been reported previously, with CADRs frequently mistaken for viral exanthems or parasitic infestations, especially in primary care settings [15]. Immune-mediated ADRs, often referred to as drug allergies, account for less than 20% of all ADRs. These reactions are typically unpredictable and classified as Type B reactions. Among them, Type I (IgE-mediated) and Type IV (T-cell-mediated) hypersensitivity reactions are the most clinically relevant, as described in the Gell and Coombs classification [17].

Although IgE- and T-cell-mediated mechanisms are well recognized, the precise pathophysiology of antibiotic-induced allergic reactions, particularly with amoxicillin, remains incompletely understood. Beyond classical immune pathways, the pharmacological interaction with immune receptors (p-i) concept proposes that certain antibiotics can directly activate T-cell receptors or major histocompatibility complex (MHC) molecules without prior sensitization, thereby triggering immune responses [18].

In the present case, causality assessment using the Naranjo Adverse Drug Reaction Probability Scale categorized the reaction as probable [12]. Severity assessment using the Modified Hartwig and Siegel scale classified the reaction as moderate, as it required medical intervention without hospitalization or permanent harm [13]. Preventability assessment using the Schumock and Thornton criteria suggested that the reaction was probably preventable, given the availability of alternative antibiotics and the non-life-threatening nature of the initial infection [14]. Application of these structured tools enhances the objectivity and clinical relevance of ADR reporting.

This case underscores the need for heightened clinical awareness of rare and atypical cutaneous reactions associated with commonly prescribed antibiotics. Early recognition, prompt discontinuation of the offending drug, and appropriate supportive management can reduce patient morbidity and prevent unnecessary complications. Furthermore, reporting such rare presentations contributes to pharmacovigilance databases, improving the overall understanding of drug safety profiles and supporting safer prescribing practices.

Limitations

This case report has certain limitations inherent to single-patient observations. As an isolated case, the findings cannot be generalized to broader populations, nor can the true incidence or risk of amoxicillin-clavulanate-induced pityriasis rosea be determined. The diagnosis was primarily based on clinical features, temporal association, and response to drug withdrawal, without histopathological confirmation, which may limit diagnostic certainty. In addition, long-term follow-up was limited, restricting assessment of recurrence or delayed sequelae. Finally, pharmacovigilance data are dependent on spontaneous reporting and are known to underestimate the actual burden of rare ADRs.

Future directions

Future research should focus on systematic documentation of drug-induced pityriasis rosea through multicenter registries and pharmacovigilance databases to better characterize its incidence, risk factors, and clinical patterns. Prospective studies exploring immunological mechanisms underlying antibiotic-induced cutaneous reactions may help clarify pathogenesis and identify susceptible individuals. Increased clinician education and integration of ADR reporting into routine clinical workflows could further strengthen pharmacovigilance efforts. Additionally, developing clear diagnostic criteria to distinguish drug-induced pityriasis rosea from idiopathic forms may improve early recognition and management.

## Conclusions

Pityriasis rosea is an uncommon but important cutaneous adverse reaction associated with amoxicillin-clavulanate. This case underscores the diagnostic challenges posed by atypical drug-induced skin eruptions and highlights the importance of careful drug history, early recognition, and prompt discontinuation of the offending agent. Timely symptomatic management resulted in complete clinical recovery. Reporting rare ADRs such as this contributes valuable information to pharmacovigilance systems and supports safer prescribing practices for widely used antibiotics.

## References

[1] Kaur M, Mongia AK, Joseph G, Bhatti N, Badyal DK (2025). Ondansetron-induced dystonia: an uncommon case report. Natl J Pharmacol Ther.

[2] Abhilasha P, Bhatti N, Joseph G, Badyal DK (2024). Sodium valproate-induced hyperammonemia: a case series in a tertiary care hospital. Cureus.

[3] Joseph G, Bhatti N, Badyal DK, Kaur P (2025). Stevens-Johnson syndrome: an adverse drug reaction with various drugs. Indian J Physiol Pharmacol.

[4] Lu Y, Zhou L, Zou Y (2024). Antibiotic-induced severe cutaneous adverse reactions: a single-center retrospective study over ten years. Front Immunol.

[5] Pandey N, Cascella M (2023). Beta-lactam antibiotics. StatPearls [Internet].

[6] Matho A, Mulqueen M, Tanino M (2018). High-dose versus standard-dose amoxicillin/clavulanate for clinically-diagnosed acute bacterial sinusitis: a randomized clinical trial. PLoS One.

[7] Salvadori M, Audino E, Venturi G, Garo ML, Salgarello S (2019). Antibiotic prescribing for endodontic infections: a survey of dental students in Italy. Int Endod J.

[8] Zhang S, Dong T, Xian J (2024). Association between penicillin allergy labels and serious adverse events in hospitalized patients: a systematic review and meta-analysis. Front Pharmacol.

[9] Huang H, Li L, Wu M (2023). Antibiotics and antibiotic-associated diarrhea: a real-world disproportionality study of the FDA adverse event reporting system from 2004 to 2022. BMC Pharmacol Toxicol.

[10] Evans J, Hanoodi M, Wittler M (2024). Amoxicillin clavulanate. StatPearls [Internet].

[11] (2025). VigiAccess. https://www.vigiaccess.org/.

[12] Naranjo CA, Busto U, Sellers EM (1981). A method for estimating the probability of adverse drug reactions. Clin Pharmacol Ther.

[13] Hartwig SC, Siegel J, Schneider PJ (1992). Preventability and severity assessment in reporting adverse drug reactions. Am J Hosp Pharm.

[14] Pirmohamed M, James S, Meakin S (2004). Adverse drug reactions as cause of admission to hospital: prospective analysis of 18 820 patients. BMJ.

[15] Leung AK, Lam JM, Leong KF, Hon KL (2021). Pityriasis rosea: an updated review. Curr Pediatr Rev.

[16] Chuh A, Lee A, Zawar V, Sciallis GF, Kempf W (2015). Pityriasis rosea-like eruptions: a review of causes and clinical features. Indian J Dermatol Venereol Leprol.

[17] Pavlos R, Mallal S, Ostrov D, Buus S, Metushi I, Peters B, Phillips E (2015). T cell-mediated hypersensitivity reactions to drugs. Annu Rev Med.

[18] Pichler WJ (2008). The p-i concept: pharmacological interaction of drugs with immune receptors. World Allergy Organ J.

